# Formulation and Evaluation of Capsules From *Azadirachta indica* and *Khaya senegalensis* for Malaria Treatment

**DOI:** 10.1155/jotm/8366410

**Published:** 2026-04-20

**Authors:** Winifred Naa Adoley, Raphael Johnson, Mariam El Boakye-Gyasi, Frederick William Akuffo Owusu, Lawrence Michael Obeng, Antwi Osei-Asibey, Kofi Acheampong Asamoa Mensa, Felix Kwame Zoiku

**Affiliations:** ^1^ Department of Pharmaceutics, Faculty of Pharmacy and Pharmaceutical Sciences, Kwame Nkrumah University of Science and Technology, Kumasi, Ghana, knust.edu.gh; ^2^ Department of Pharmaceutics, Faculty of Pharmacy and Pharmaceutical Sciences, University of Health and Allied Sciences, Ho, Ghana, uhas.edu.gh; ^3^ Department of Epidemiology, Noguchi Memorial Institute for Medical Research, College of Health Sciences, University of Ghana, Legon, Accra, Ghana, ug.edu.gh

## Abstract

Malaria is a major public health problem, prevalent in Africa and endemic in Ghana. Besides the use of artemisinin combination therapy, plant medicine is well‐recognised and accepted in Ghana. Decoctions of *Azadirachta indica* (AI) leaves and *Khaya senegalensis* (KS) stem bark are traditionally used for treating malaria. However, these decoctions are bitter, bulky and unstable compared to solid dosage forms. This study sought to formulate decoctions from these plants into capsules to tackle these setbacks. The maximum wavelength of absorption and the amount per dose of both plants’ decoctions (120 mL) were determined. Four absorbents (bentonite, magnesium carbonate, microcrystalline cellulose and kaolin) at four concentrations (25, 50, 75 and 100 mg/dose) were used to formulate the decoctions into granules. The granules were assessed to select the best extract‐absorbent granules for encapsulation and quality assessment. Antiplasmodial activity and cytotoxicity of the formulated capsules were also determined. AI containing 35 mg/dose of kaolin with a percentage release of 97.31 ± 0.74% and good flow properties was encapsulated. KS containing 50 mg/dose of bentonite with a percentage release of 97.54 ± 0.88% and good flow properties was encapsulated. Formulated capsules from both plants passed all quality control tests performed, with disintegration times of 10.01 ± 0.34 and 4.48 ± 0.23 min, and cumulative drug release of 78.13 ± 0.37% and 95.63 ± 1.00%, for AI and KS, respectively. AI (IC_50_, 2.74 ± 0.70 μg/mL) and KS (IC_50_, 24.37 ± 4.19 μg/mL) had good to moderate antiplasmodial activity (IC_50_ ≤ 50 μg/mL) using the SYBR green assay. Cytotoxicity studies also indicated that both AI and KS were selective for the *Plasmodium* parasite. The formulated capsules could therefore be used as an alternative to their bitter decoctions.

## 1. Introduction

Malaria is a disease caused by a protozoan of the species *Plasmodium,* with *Plasmodium falciparum* infection being the most severe and potentially fatal of all the species [1]. It is a major public health problem that is prevalent in Africa, with an estimated 233 million cases accounting for 94% of the global malaria cases in the WHO African Region [2]. In Ghana, a malaria‐endemic country, it was ranked as the first disease that causes the most deaths and second among those that lead to premature deaths according to the Institute for Health Metrics and Evaluation [3]. Aside from the use of artemisinin‐based combination therapy (ACT) as a first‐line treatment for malaria caused by *P. falciparum* [2], the use of herbal medicine is well recognised in Ghana.

Herbal medicinal products in recent decades have seen an upsurge globally, with about 80% of the world’s population resorting to plant medicine [4]. Over 60% of Ghana’s population has been reported to make use of herbal medicinal products to fulfil primary healthcare demands [5]. Most of these herbal medicines, however, are in liquid dosage forms (decoctions, concoctions and mixtures), raising concerns about the stability, appropriateness of dosage forms and analytical control procedures [6], taste, accuracy in dosing and convenience [7]. The stem bark of *Khaya senegalensis* (mahogany) and the leaves of *Azadirachta indica* (neem) are folklorically used as antimalarials [8], and their antiplasmodial potency is reported in different literature [9–11]. However, the bitter taste and bulkiness of their decoctions create inconveniences for patients who resort to these plants for treatment [7]. To curb this problem, patients need to be provided with more favourable herbal dosage forms as a way to increase compliance to ensure maximum therapeutic effect [12], and this can be achieved by encapsulation. A capsule is a dosage form that uses a shell of gelatin into which active ingredients and/or inert substances are enclosed [13]. They are known to mask the taste of drugs, have accurate doses, are easy to carry, and are very stable. This study sought to formulate capsules from the aforementioned plants, assess their quality and evaluate their antiplasmodial activity.

## 2. Materials and Methods

### 2.1. Materials

#### 2.1.1. Plant Material

The stem bark of *K. senegalensis* (voucher number KNUST/HMI/2009/L004) and leaves of *A. indica* (voucher number KNUST/HMI/2015/B006) were harvested in March 2021 and July 2022, respectively, at Kwahu–Asakraka in the Eastern Region of Ghana and authenticated by Dr George H. Sam of the Department of Herbal Medicine, Faculty of Pharmacy and Pharmaceutical Sciences at the Kwame Nkrumah University of Science and Technology, Kumasi.

#### 2.1.2. Excipients and Other Materials

Bentonite powder, light magnesium carbonate powder, kaolin powder, microcrystalline cellulose powder, lactose, starch and talc powder (Department of Pharmaceutics, Faculty of Pharmacy and Pharmaceutical Sciences, KNUST); ammoniacal alcohol, sulphuric acid, ammonia solution, Dragendoff’s solution, hydrochloric acid, Fehling’s solutions A and B, chloroform and lead acetate (Department of Pharmacognosy, KNUST); chloroquine‐sensitive strain of *Plasmodium falciparum* (3D7); chloroquine‐resistant strain of *Plasmodium falciparum* (DD2) (Noguchi Memorial Institute for Medical Research, University of Ghana); complete medium, dimethyl sulfoxide (DMSO), lysing buffer, MTT solution [3‐(4,5‐dimethylthiazol‐2‐yl)‐2,5‐diphenyl‐2H‐tetrazolium bromide] and Triton X‐100 (Sigma Aldrich, Germany; QualiChem’s Lab Reagents, India).

#### 2.1.3. Equipment

Analytical balance (Jos. Hansen and Soehne GmbH, Hamburg, Germany), UV–visible spectrophotometer (Cecil 7200 series, England), Gallenkamp hot air oven (300 Plus series, England), Retsch laboratory test series (D‐32759 HANN, Germany), Erweka dissolution apparatus (DT6, Nr 68,045, GmbH Heusenstamm, Germany), Veego digital tablet disintegration test apparatus (VTD‐DV, 05/0721, India) and FLUOstar OPTIMA Fluorometer (BGM LABTECH, Germany) were used.

### 2.2. Method

#### 2.2.1. Plant Processing and Preparation of Decoctions

The plants were air‐dried for 7 days, coarsely powdered and stored in a well‐closed container till further use. Decoctions of both plants were prepared with 300 g of the processed plant materials in 9 L of water, simmered to 6 L and decanted and sieved with a calico strainer [8, 11].

#### 2.2.2. Determination of Amount per Dose of Decoctions

An amount of 120 mL of the decoctions of both plants, which equals a dose [8, 14], was evaporated to dryness in a hot air oven at 60°C, and the weight of the dried extract of the decoction was determined. This was done in triplicate for each of the plants.

#### 2.2.3. Maximum Wavelength of Absorption (λ_max_) and Calibration Curves

Serial dilutions (0.0001, 0.001, 0.01 and 0.1% w/v) of 1% w/v stock solutions of *K. senegalensis* (KS) stem bark and *A. indica* (AI) leaf extracts were prepared, and their maximum wavelength of absorption was scanned using a UV–visible spectrophotometer. Concentrations of 0.001% w/v of KS and 0.01% w/v of AI gave λ_max_ of 257 and 535 nm, respectively. The absorbances of solutions of different concentrations of both plant extracts were determined at their λ_max_ to plot calibration curves for subsequent analysis.

#### 2.2.4. Phytochemical Screening of Plant Extract

The dried extract of KS and AI was screened to ascertain the presence of phytochemical constituents following standard procedures [15, 16].

#### 2.2.5. Preparation and Evaluation of Granules

Decoctions of both plants were evaporated to concentrated extracts in a hot air oven at 60°C and mixed individually with bentonite, magnesium carbonate, microcrystalline cellulose and kaolin to form a uniform paste at concentrations of 25, 50, 75 and 100 mg per dose of *K. senegalensis* and 17.5, 35, 52.5 and 70 mg per dose of *A. indica*. Further drying was continued to obtain a completely dried extract‐absorbent mass and screened through a Retsch analytical sieve 60 (aperture 250 μm) to obtain granules of both plants. The flow properties of the formulated granules were determined by the angle of repose, Carr’s compressibility index and Hausner ratio methods.

#### 2.2.6. Release Profile of Granules and Dissolution Testing for Capsules

The release profiles of the formulated granules were determined using an Erweka dissolution apparatus and the United States Pharmacopoeia standards. The experimental conditions employed were as follows: dissolution medium: 900 mL of distilled water; paddle speed: 50 revolutions per minute; sampling times: 5, 10, 15, 30, 45 and 60 min; temperature: 37 ± 0.5°C. Aliquots of 10 mL were withdrawn at the sampling times and replaced with a fresh dissolution medium. The withdrawn samples were filtered and diluted appropriately with distilled water. The absorbances of the samples were measured by UV spectrophotometry at 257 and 535 nm for *K. senegalensis* and *A. indica,* respectively, and the concentration of the extracts was determined using the equations of the calibration curves plotted. The cumulative percentage release of each sample was calculated, and a dissolution profile was plotted.

#### 2.2.7. Encapsulation of Granules

Starch (5% w/w), lactose and talc (1% w/w), which served as a disintegrant, a diluent and a lubricant, respectively, were added to the *K. senegalensis* and *A. indica* extract‐absorbent granules. Using a semiautomated capsule machine, the granules were encapsulated into a size 0 hard capsule shell.

#### 2.2.8. Quality Assessment of Formulated Capsules

The formulated capsules were assessed for quality using the uniformity of weight, disintegration and dissolution tests described in the United States Pharmacopoeia [17].

##### 2.2.8.1. Uniformity of Weight

Randomly, twenty formulated capsules of both plants were selected. The weight of one intact capsule of the selected capsule was determined using an analytical balance. The capsule was carefully opened, the contents were removed, and the weight of the capsule shell was determined. The difference between the intact capsule and the empty shell was calculated to find the net weight of the content. The procedure was repeated for the nineteen other capsules of each of the plants; the average weight of the content was determined, and the percentage deviation was calculated to assess the uniformity of weight.

##### 2.2.8.2. Disintegration Test

The water bath of the disintegration test apparatus was set to a temperature of 37 ± 2°C. The beaker was filled with distilled water to the 1000 mL mark and suspended in the main bath. One capsule was placed into each of the six tubes of the basket, and the apparatus was operated until all six capsules disintegrated, leaving only fragments of the capsule shells. The time taken was noted, the test was then repeated two more times for both plants, and the average time was determined as the disintegration time.

##### 2.2.8.3. Dissolution Test

The dissolution profile for formulated capsules was determined with the same procedure and experimental conditions as employed in the release profile determination of the granules.

#### 2.2.9. In Vitro Antiplasmodial Activity

##### 2.2.9.1. Parasite Culturing

The antiplasmodial activity of the formulated capsules of both plants was tested against 3D7 and DD2 using a modified method of Trager and Jensen [18]. The parasites were maintained in vitro in an atmosphere of 90% N_2_, 5% CO_2_ and 5% O_2_ at 37°C in a CM (10.44 g/L RPMI 1640, 5.94 g/L HEPES, 5 g/L AlbuMAX II, 50 mg/L hypoxanthine and 2.1 g/L sodium bicarbonate). The parasites were cultured in O^+^ RBCs and were maintained in an incubator with daily media change until more than 5% parasitaemia was acquired. The culture was synchronised with 5% sorbitol at the ring stage and allowed to grow to > 5% parasitaemia. A parasite suspension of 2% haematocrit from uninfected blood and 1% parasitaemia was mixed in 11 mL of CM for plating.

##### 2.2.9.2. Extract Plating and Assay

A stock solution of 1000 μg/mL of the content of the formulated capsules of both plants was individually prepared with 0.5% DMSO, vortexed, filtered through a 0.2‐μm pore filter and further diluted to nine solutions (100, 50, 25, 12.5, 6.25, 3.13, 1.56, 0.78 and 0.39 μg/mL). An aliquot of each of the solutions was plated in duplicates in a 96‐well coastal plate, and artesunate (15 ng/mL) was serially diluted and plated alongside the samples as a standard drug. One hundred parasites with 2% haematocrit and 1% parasitaemia were added to each treated well, starting from the 2^nd^ to the 10^th^ well, with the 11^th^ well as the negative control (parasite suspension). The plates were arranged in a modular chamber and gassed for 5 min with a gas mixture of 5% O_2_, 5% CO_2_ and 90% N_2_ and then kept at 37°C for 72 h. The cultures were then treated with 100 μL lysing buffer containing SYBR green in each well, thoroughly and gently mixing. The plates were then incubated in the dark for 30–60 min before measurement at 470 and 520 nm using the FLUOstar OPTIMA fluorometer. The concentration of inhibition (IC_50_) was estimated from dose–response curves by nonlinear regression analysis using GraphPad Prism software (GraphPad Software, Version 7.0, San Diego, CA, USA).

##### 2.2.9.3. Cytotoxicity Assay of Capsules

The toxicity of the formulated capsules of both plants to RBCs was tested using a modified version of the tetrazolium‐based colourimetric technique by Ayisi et al. [19]. Approximately 0.1 mL of each diluted sample (6.25–100 μg/mL) was put (in triplicate) into a 96‐well microtiter plate. A volume of 0.1 mL of uninfected erythrocytes was added to each well and incubated for 3 days at 37°C in a humidified incubator (5% O_2_ and CO_2_). A volume of 20 μL of 7.5 mg/mL MTT (in phosphate‐buffered saline) solution was added to each well, and the plate was incubated for another 2 h. A volume of 0.2 mL of Triton X‐100 in acidified isopropanol was then added to each well to dissolve any formazan produced. The plates were then maintained at room temperature in the dark for 24 h before the optical densities were measured at 570 nm in the microplate reader. The concentrations at which a 50% cytotoxic effect occurred (CC_50_) were determined using GraphPad Prism (Version 9.5.1). The CC_50_ values were compared to standard values. The CC_50_ value was also compared to the IC_50_ to find the selective index of the formulated capsules of both plants.

## 3. Results and Discussion

### 3.1. Preparation and Determination of Amount per Dose of Decoction

The use of plant medicines in the management of malaria in Ghana is well acknowledged. However, most herbal medicines sold on the market are known to be very bitter and bulky. From the study, it was established that a dose of the decoction of both *K. senegalensis* and *A. indica* was 120 mL (one teacup), which is to be taken three times daily for about 7 days [8, 14]. The decoction packaged in 500 mL bottles suggests that a patient needs to take about five to six bottles of the decoction to ensure complete recovery. This may discourage a patient from acquiring herbal remedies, considering the inconveniences involved in consuming this large number of bitter medicines and carrying huge bottles around, coupled with concerns about stability and analytical control procedures. Solid dosage forms have accurate dosing, are more stable and aesthetically appealing, and are less susceptible to microbial growth compared with liquid dosage forms [20]. In an attempt to curb this setback, this work sought to develop two plants with established antimalarial properties into suitable, portable solid dosage forms (capsules).

To establish a good formula for the capsule formulation, it was expedient to determine the dried extract equivalence of a dose of their decoctions. The drying of the aqueous decoction of both plants yielded a gummy‐like substance, which was very highly hygroscopic. This would make the formulation into a solid dosage form very difficult; hence, the use of absorbents, as also employed by [6, 12, 21], was considered. The amount per dose of decoctions of KS and AI was 0.800 ± 0.002 and 0.830 ± 0.004 g, respectively.

### 3.2. Calibration Curve

The calibration curve (Figure 1) showed a linear relationship between the concentration of the extract and absorbance, with the equation of the line as *y* = 65.464*x* + 0.0065 and the coefficient of determination (*R* ^2^) as 0.9991 for KS and *y* = 9.1925*x* − 0.0403 and the coefficient of determination (*R* ^2^) is 0.9994 for AI. The maximum wavelength of absorption (λmax) measured for KS and AI was used as a marker of the constituents for content evaluation. The calibration curves were used to quantify the active constituents present in the formulation responsible for their efficacy.

FIGURE 1Calibration curve of *K. senegalensis* (a) and *A. indica* (b).

**Figure:**
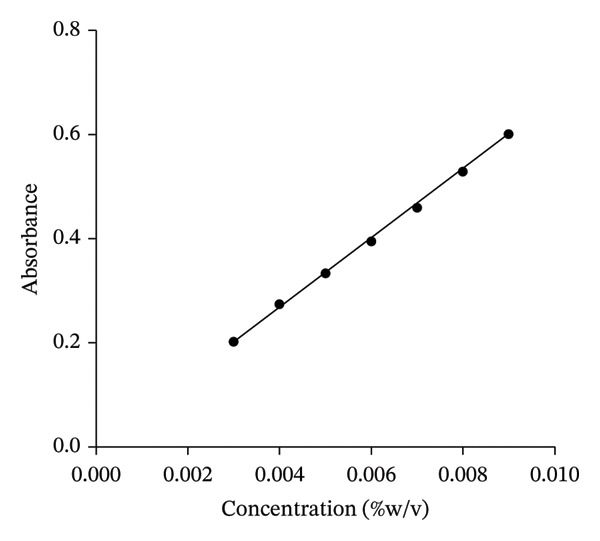
(a)

**Figure:**
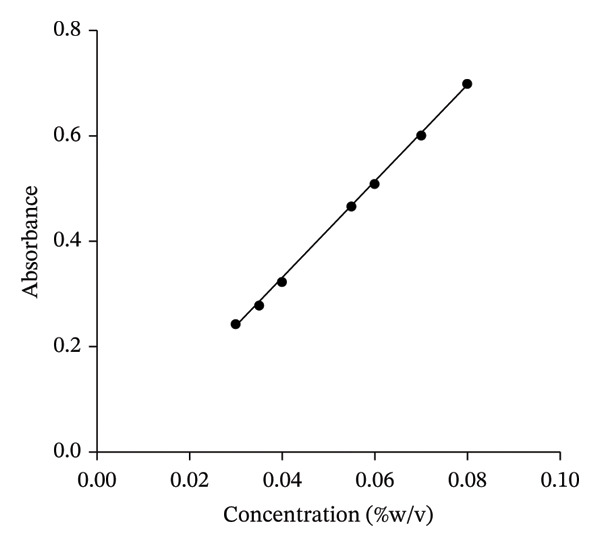
(b)

### 3.3. Phytochemical Screening

Secondary metabolites found in plants account for their therapeutic activity [8, 11]. Phytochemical screening is important to ensure the authenticity and easy identification of the plants harvested. All phytoconstituents screened for were present in both plants, except phytosterol, which was absent in KS (Table 1), and these results fulfilled the standards of the Ghana Herbal Pharmacopoeia.

**TABLE 1:** Phytochemical screening of KS and AI dried extract.

Secondary metabolites	Inference for KS	Inference for AI
Alkaloids	+	+
Coumarins	+	+
Flavonoids	+	+
Phytosterols	−	+
Reducing Sugars	+	+
Saponins	+	+
Tannins	+	+
Triterpenols	+	+

*Note:* + (present); −(absent).

### 3.4. Flow Properties of Granules

An important parameter in tablet and capsule formulation is the flowability of the powder or granules. It is very useful in the clinical application of drug uniformity as a dosage form [22] and can significantly impact manufacturing efficiency and product quality. Carr’s index, Hausner ratio and angle of repose measure inter‐particulate friction and cohesion, as well as the effective packing of granules during capsule filling [23]. These parameters indicate poor flowability and rough and irregular particles when high and vice versa. The results showed that the flow properties of the granules of both plant extracts with different absorbents at different concentrations were generally good to passable compared to the standard scale of flowability (Tables 2 and 3). However, comparatively, the granules of KS had better flowability than the granules of AI.

**TABLE 2:** Flow properties of KS granules.

Absorbent	Weight per dose (mg)	Angle of repose (°)	Hausner ratio	Carr’s index (%)
Bentonite	25	32	1.25	20.0
	50	29	1.17	14.3
	75	28	1.18	15.4
	100	28	1.17	14.3

Magnesium carbonate	25	30	1.25	20.0
	50	28	1.17	14.3
	75	31	1.25	20.0
	100	28	1.15	13.3

Microcrystalline cellulose	25	32	1.23	18.8
	50	31	1.23	18.8
	75	28	1.23	18.8
	100	30	1.25	20.0

Kaolin	25	30	1.23	25.0
	50	30	1.23	14.3
	75	31	1.23	15.4
	100	27	1.25	20.0

*Note:* Angle of repose: 25°–30° ⟶ excellent. 31°–35° ⟶ good. Hausner ratio: 1.12–1.18 ⟶ good. 1.19–1.25 ⟶ fair. Carr’s index: 11%–15% ⟶ good. 16%–20% ⟶ fair. 21%–25% ⟶ passable.

**TABLE 3:** Flow properties of AI granules.

Absorbent	Weight per dose (mg)	Angle of repose (°)	Hausner ratio	Carr’s index (%)
Bentonite	17.5	29	1.23	18.8
	35.0	33	1.25	20.0
	52.5	32	1.33	25.0
	70.0	33	1.17	14.3

Magnesium carbonate	17.5	32	1.31	23.5
	35.0	33	1.23	18.8
	52.5	32	1.33	25.0
	70.0	32	1.21	17.6

Microcrystalline cellulose	17.5	32	1.22	18.8
	35.0	32	1.31	23.5
	52.5	30	1.14	12.5
	70.0	32	1.23	18.8

Kaolin	17.5	31	1.25	20.0
	35.0	32	1.17	14.3
	52.5	34	1.31	23.5
	70.0	31	1.31	23.5

### 3.5. Release Profile of Formulated Granules

The use of pharmaceutical absorbents as excipients enhances the formulation of liquid dosage forms into solid dosage forms. Notwithstanding, absorbents tend to alter the bioavailability of drugs [24]. The ability of the different absorbents to release the plant extracts at different concentrations was investigated using the dissolution procedure to find the absorbent with the best release profile (Figures 2 and 3). The United States Pharmacopoeia [17] states that for the dissolution of botanicals, not less than 75% of the drug must be dissolved in 60 min. From the results, the percentage release of KS extract from the bentonite increased with increasing concentration, stipulating that the higher the concentration of the absorbent, the better the release of the extract from the absorbent. At all the concentrations, the absorbent released more than 75% of the extract at 60 min and had better release compared to the extract only; hence, they all passed the test. The percentage release of the KS extract from magnesium carbonate decreased remarkably with increasing concentrations of the absorbent per dose, indicating that at high concentrations of the absorbent, the extract tends to bind tightly to the absorbent, making it difficult to release the extract. Microcrystalline cellulose at different concentrations released more than 75% of the KS extracts within 60 min; they all passed the test, with their release profiles being better than that of the extract only. The percentage release of the extract from kaolin at different concentrations was more than 75% at 60 min, also passing the test. However, there was a considerable decrease in the release of the concentration of 100 mg per dose compared to the dissolution of the extract, only specifying that at a concentration beyond 75 mg, there is a possible reduction in the ability of kaolin to release the extract.

FIGURE 2Release profile of KS granules with bentonite (a), magnesium carbonate (b), microcrystalline cellulose (c) and kaolin (d) at different concentrations.

**Figure:**
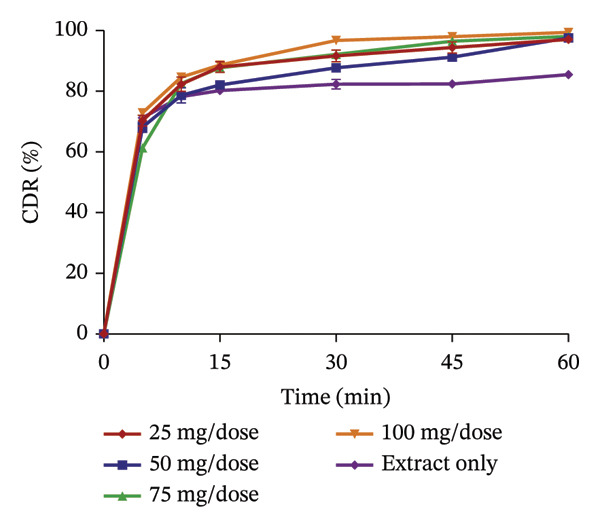
(a)

**Figure:**
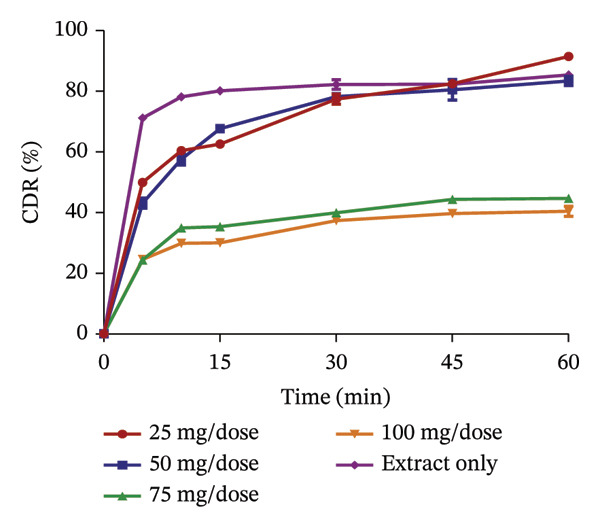
(b)

**Figure:**
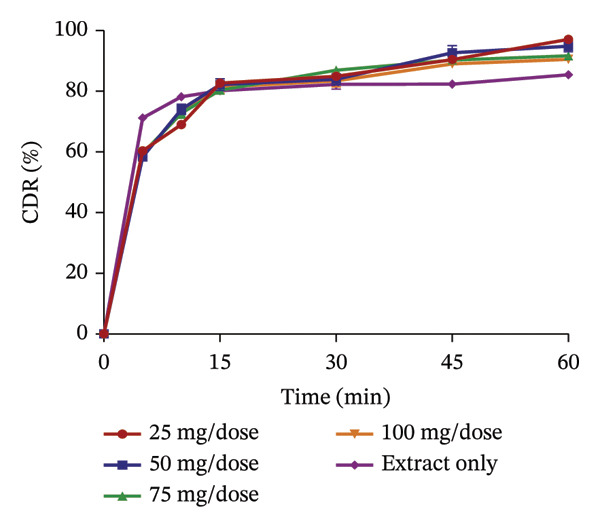
(c)

**Figure:**
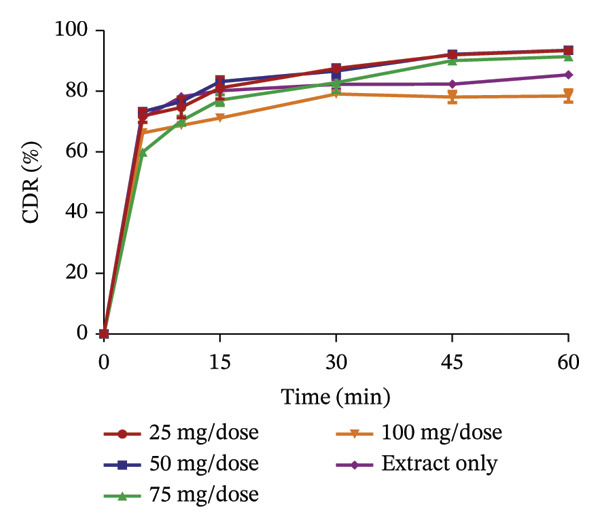
(d)

FIGURE 3Release profile of AI granules with bentonite (a), magnesium carbonate (b), microcrystalline cellulose (c) and kaolin (d) at different concentrations.

**Figure:**
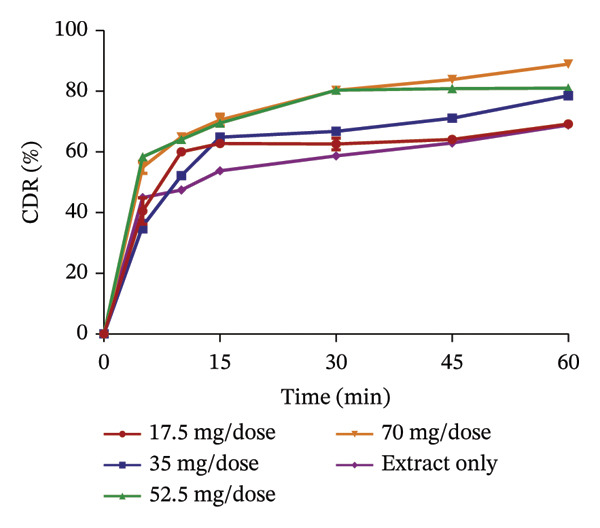
(a)

**Figure:**
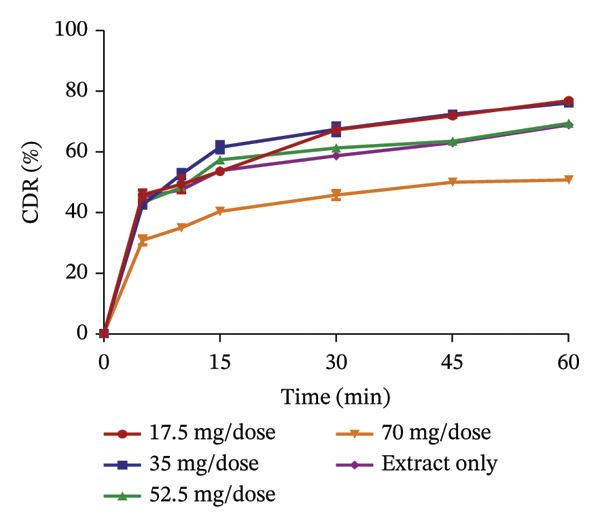
(b)

**Figure:**
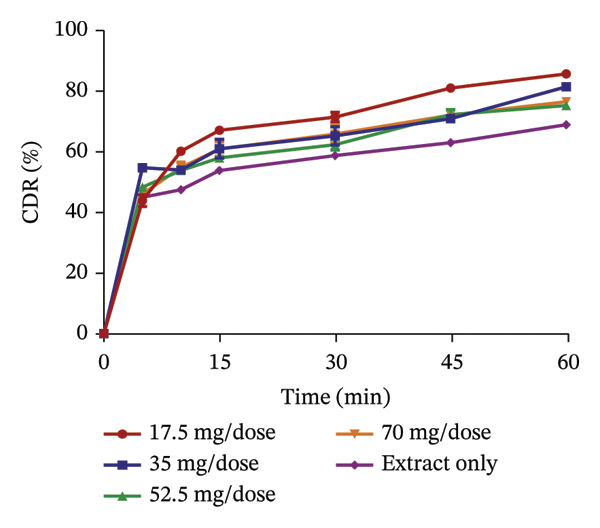
(c)

**Figure:**
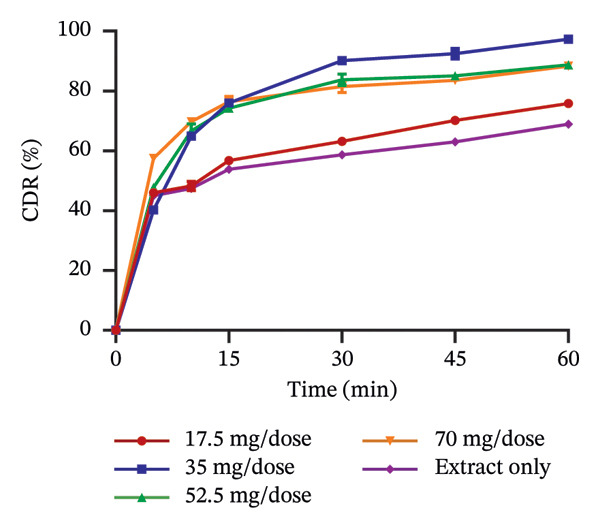
(d)

The release profiles of AI extracted from the various absorbents (Figure 3) were similar to that of KS. Bentonite at different concentrations had a release profile significantly greater than that of the extract only, with an increase in percentage release as concentrations increased. However, granules with 17.5 mg per dose of the absorbent failed the test, having a release of the extract of less than 75% at 60 min, with the rest passing the test. This shows that an increase in the concentration of bentonite enhances the release of the extract from the absorbent. Magnesium carbonate as an absorbent had a relatively poor release profile, as extracts with 17.5 and 35 mg of absorbent per dose only passed the test with a percentage release above 75% at 60 min. The increase in the concentration of this absorbent relatively decreased the release of the AI extract from the absorbent. Compared to the extract only, granules with 70 mg per dose had the lowest percentage release. The release profiles of the AI extract from microcrystalline cellulose were all better compared to that of the extract only and passed the test. However, an increase in the concentration showed a decrease in the release of the extract from the absorbent. Kaolin had the best release profile compared to the other absorbents. The different concentrations of kaolin showed better results compared to the extract only. Also, 35 mg of kaolin per dose of AI gave the best release with 97.31 ± 0.74% of the drug released. There was, however, a considerable decrease in the rate of release at 52.5 and 70 mg per dose of the absorbent.

The dissolution of capsules is affected by the particle size of the powder or granules used in the formulation [23]. Both plant extracts formulated with different absorbents at different concentrations were all passed through a sieve with a number 60 (approximately 250 µm), giving them a uniform and relatively small size. This also enhanced the dissolution of the granules. Considering the release profile of all the absorbents at different concentrations of KS and AI, their flow properties (angle of repose, Hausner ratio and Carr’s index), ease of scraping and percentage loss of the granules (which were preliminary studies to select the best extract‐absorbent granules for both plants), the KS extract with 50 mg of bentonite per dose and the AI extract with 35 mg per dose of kaolin were selected for encapsulation.

### 3.6. Quality Control Tests

**FIGURE 4:**
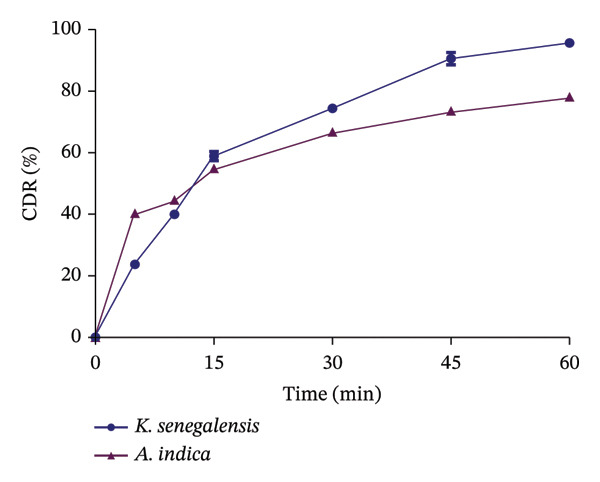
Dissolution profile of formulated *K. senegalensis* and *A. indica* capsules.

Quality assessment is very important when it comes to the formulation of drugs. The quality of the formulated capsules was assessed using the uniformity of weight, the disintegration test and the dissolution test. A uniformity of weight test is used to ensure that there is little or no variation in the weight of the formulated dosage form. According to the United States Pharmacopoeia, for botanicals (classified under dietary supplements), capsules pass the weight variation test if the content difference of not more than two capsules out of the twenty capsules individually weighed is greater than 10% of the average net content and none is greater than 25% of the average net weight of the twenty capsules. From the percentage deviation calculated for the individual capsules, both capsules of KS and AI passed the test, as all twenty capsules of both plants were within the stated limit (Tables 4 and 5). The use of the hand‐operated machine also enhanced the uniformity of the capsules since a similar amount of fill material is ensured to fill each capsule. The disintegration test suggests how a drug breaks down in solution when taken into the gastrointestinal tract. The average disintegration time for KS capsules was 4.48 ± 0.23 min, and that of AI capsules was 10.01 ± 0.34 min (Table 6), passing the test (less than 30 min). This shows that the capsules can be broken down appropriately in the gastrointestinal tract for maximum efficacy [17]. The dissolution test is a standardised method for estimating the rate of drug release from a given dosage form to predict the release of drugs in vitro. There was a release of more than 75% (Figure 4) of the content of the capsules at 60 min (95.63 ± 1.00% for KS and 78.13 ± 0.37% for AI), indicating that both capsules passed the test. This suggests that the capsules can be dissolved in the fluid of the gastrointestinal tract to release the active constituents for maximum absorption and circulation.

**TABLE 4:** Uniformity of weight of AI capsules.

Capsule number	Net weight	Deviation (%)
1	0.549	−0.18
2	0.562	−2.55
3	0.549	0.18
4	0.538	1.82
5	0.550	−0.36
6	0.559	−2.01
7	0.544	0.73
8	0.542	1.09
9	0.555	−1.28
10	0.548	0.00
11	0.553	−0.91
12	0.535	2.37
13	0.546	0.36
14	0.555	−1.28
15	0.544	0.73
16	0.541	1.28
17	0.559	−2.01
18	0.537	2.01
19	0.543	0.91
20	0.548	0.00

**TABLE 5:** Uniformity of weight of KS capsules.

Capsule number	Net weight	Deviation (%)
1	0.518	0.19
2	0.495	4.62
3	0.532	−2.50
4	0.507	2.31
5	0.545	−5.01
6	0.516	0.58
7	0.498	4.05
8	0.540	−4.05
9	0.516	0.58
10	0.514	0.96
11	0.494	4.82
12	0.511	1.54
13	0.538	−3.66
14	0.539	−3.85
15	0.543	−4.62
16	0.496	4.43
17	0.511	1.54
18	0.529	−0.192
19	0.530	−2.11
20	0.508	2.11

**TABLE 6:** Disintegration test of KS and AI capsules.

Determination	*K. senegalensis*	*A. indica*
	Time (min)	Time (min)
1	4.48	10.35
2	5.11	9.27
3	4.25	10.01
Average	4.48 ± 0.23	10.01 ± 0.34

### 3.7. Antiplasmodial Activity and Cytotoxicity Studies

Although there have been several studies to ascertain the antimalarial activities of *K. senegalensis* and *A. indica*, it was important to determine the antimalarial activity of the formulated capsules to ensure that the processes used in the formulation did not affect the antimalarial properties of the capsules. The in vitro antiplasmodial activity of both plants using the SYBR green assay confirmed the antiplasmodial activity of the plant extracts as reported by Gouissi et al. [25] and Saidu et al. (2011) for KS and by Aladesanmi et al. [26] and Akin‐Osanaiya et al. [27] for AI. According to Cudjoe et al. [28], using the standard 72‐h test, herbal extracts’ antiplasmodial activity against asexual disease‐causing parasites has been categorised as good (IC_50_ values less than 10 μg/mL), moderate (IC_50_ values from 10 to 50 μg/mL), low (IC_50_ values from 50 to 100 μg/mL) and inactive (IC_50_ values > 100 μg/mL). The resulting IC_50_ of both plants’ extract only and capsules showed that AI capsules and extract only and KS pure extract had good activity against the strains, while KS capsules had moderate activity against the same lab strains (Table 7). The standard drug (artesunate), however, showed better activity than the plant extracts (IC_50_: 0.0013 ± 0.0001). Cytotoxicity of the formulated capsules and plant extracts only was also investigated to determine the concentrations at which the desired effects can be achieved without inducing toxic effects on the red blood cells. From the results, the selectivity index of the AI extract was similar to that of the artesunate control, with SI greater than 100. KS capsules had the least SI (9.01), followed by the extract only (53.76) and the AI extract only (73.07) for the 3D7 lab strain. Drugs with selectivity index values greater than or equal to 10 are generally considered to be active; thus, SI serves as a guide for drug development [29]. The higher the SI, the more selective and effective the drug is against the *Plasmodium* parasite and the safer the drug is for normal cells [30]. It can therefore be deduced that the KS capsules were less selective for *P. falciparum* compared to AI. Comparatively, from the results, AI had better antiplasmodial activity than KS.

**TABLE 7:** IC_50_ values of samples on the 3D7 lab strain of *Plasmodium*.

Drug/extract	IC_50_ values on 3D7 (μg/mL)	IC_50_ values on DD2 (μg/mL)	CC_50_ values (μg/mL)	Selectivity index (3D7)
AI extract	1.52 ± 0.09	2.42 ± 0.29	111.0594	73.07
AI capsule	1.20 ± 0.21	2.74 ± 0.70	165.1664	> 100
KS extract	2.59 ± 0.76	5.10 ± 1.45	139.2442	53.76
KS capsule	12.08 ± 1.08	24.37 ± 4.19	108.8237	9.01
Artesunate	0.0013 ± 0.0001	0.0017 ± 0.0002	114.9053	> 100

## 4. Conclusion

KS and AI decoctions were successfully formulated into capsules and passed the uniformity of weight, disintegration and in vitro dissolution tests. The formulated capsules may therefore be used in place of their decoctions for the treatment of malaria.

## Author Contributions

Winifred Naa Adoley—study concept and design, data collection, analysis and interpretation of results and draft manuscript preparation.

Raphael Johnson—study concept and design, analysis and interpretation of results and draft manuscript preparation.

Mariam El Boakye‐Gyasi—study concept and design, and analysis and interpretation of results.

Frederick William Akuffo Owusu and Felix Kwame Zoiku—analysis and interpretation of results.

Lawrence Michael Obeng, Antwi Osei‐Asibey and Kofi Acheampong Asamoa Mensa—data collection.

## Funding

No funding was received for this manuscript.

## Disclosure

All authors reviewed the results and approved the final version of the manuscript.

## Conflicts of Interest

The authors declare no conflicts of interest.

## Data Availability

All data obtained to support the findings of this study are included in the article and are also available from the corresponding author upon request.

## References

[1] Tuteja R. , Malaria-An Overview, FEBS Journal. (2007) 274, no. 18, 4670–4679, 10.1111/j.1742-4658.2007.05997.x, 2-s2.0-34548444241. 17824953

[2] World Health Organisation , World Malaria Report 2020-20 Years of Global Progress & Challenges, 2020.

[3] Ghana , Institute for Health Metrics and Evaluation, 2019.

[4] Ekor M. , The Growing Use of Herbal Medicines: Issues Relating to Adverse Reactions and Challenges in Monitoring Safety, Frontiers in Neurology. (2014) 4, 10.3389/fphar.2013.00177, 2-s2.0-84893445474. PMC3887317 24454289

[5] Abbiw D. , Agbovic T. , and Akuetteh B. , Conservation and Sustainable Use of Medicinal Plants in Ghana: Conservation Report, 2002.

[6] Johnson R. , Bayor M. T. , and Annan K. , Release Profile of Extracts of *Bridelia ferruginea* Leaf and *Canthium glabriflorum* Stem Bark From Different Absorbents, 2010, https://www.ijpsr.com.

[7] Osei-Asare C. , Owusu F. W. A. , Entsie P. , Annan A. K. , Gyamaa R. A. , and Amenuke E. M. , Formulation and in Vitro Evaluation of Oral Capsules From Liquid Herbal Antimalarials Marketed in Ghana, Journal of Tropical Medicine. (2021) 2021, no. 1, 6694664–6694669, 10.1155/2021/6694664. 33531912 PMC7834817

[8] Ghana Herbal Pharmacopoeia , Science and Technology Policy Research Institute (STEPRI), 2007, 2nd edition, Ghana Herbal Pharmacopoeia.

[9] Isaha A. B. , Ibrahim Y. K. E. , and Iwalewa E. O. , Evaluation of the Antimalarial Properties and Standardisation of Tablets of *Azadirachta indica* (Meliaceae) in Mice, Phytotherapy Research. (2003) 17, no. 7, 807–810, 10.1002/ptr.1231, 2-s2.0-0041474574. 12916083

[10] Saidu J. , Adoum O. A. , and Mukhtar M. D. , Screening of *Cassia singuaena*, *Commiphora kerstingii*, and *Khaya senegalensis* for Brine Shrimp Lethality and Antiplasmodium Activity, ChemSearch Journal. (2011) 2, no. 1-2, 50–52.

[11] West African Herbal Pharmacopoeia, 2013, https://www.wahooas.org.

[12] Kumadoh D. , Adotey J. , Ofori-Kwakye K. , Kipo S. L. , Prah T. , and Patterson S. , Formulation of Oral Capsules from Asena Herbal Decoction, Used Traditionally in Ghana for the Treatment of Arthritis, 2014.

[13] Allen L. V. and Ansel H. C. , Ansel’s Pharmaceutical Dosage Forms and Drug Delivery System, 2014, Lippincott Williams and Wilkins.

[14] Remington J. P. , Remington: The Science and Practice of Pharmacy, 2006, 1, Lippincott Williams & Wilkins.

[15] Khandelwal K. , Practical Pharmacognosy, 2008, Pragati Books Pvt. Ltd.

[16] Evans W. C. , Trease and Evans’ Pharmacognosy, 2009, Elsevier Health Sciences.

[17] United States Pharmacopoeia , The United States Pharmacopoeial Convention, 2015, 38th edition, United States Pharmacopoeia.

[18] Trager W. and Jensen J. B. , Human Malaria Parasites in Continuous Culture, Science. (1976) 193, no. 4254, 673–675, 10.1126/science.781840, 2-s2.0-0017311840. 781840

[19] Ayisi N. K. , Appiah-Opong R. , Gyan B. , Bugyei K. , and Ekuban F. , Plasmodium Falciparum: Assessment of Selectivity of Action of Chloroquine, *Alchornea cordifolia*, *Ficus polita*, and Other Drugs by a Tetrazolium-Based Colourimetric Assay, Malaria Research and Treatment. (2011) 2011, no. 1, 10.4061/2011/816250. PMC3265290 22312574

[20] Dudhat K. R. , The Overview of Oral Solid Dosage Forms and Different Excipients Used for Solid Dosage Formulation, Global Academic Journal of Pharmacy and Drug Research. (2022) 4, no. 3, 66–72, 10.36348/gajpdr.2022.v04i03.003.

[21] Kumadoh D. , Adotey J. , Ofori-Kwakye K. , Kipo S. L. , Prah T. , and Patterson S. , Development of Oral Capsules From Enterica Herbal Decoction–a Traditional Remedy for Typhoid Fever in Ghana, Journal of Applied Pharmaceutical Science. (2015) 5, no. 4, 083–088, 10.7324/japs.2015.50414, 2-s2.0-84928735700.

[22] Shaukat Mahmud S. M. , Rizwani G. H. , Huma Shareef H. S. , Shahnaz Gauhar S. G. , Sumaira Ishaq S. I. , and Rehana Perveen R. P. , Development of Standardized Formulation of Mono Herbal (250 mg) Capsule, International Journal of Herbal Medicine. (2013) 1, no. 4, 44–49.

[23] Aulton M. E. and Taylor K. M. G. , Aulton M. E. and Taylor K. M. G. , Aulton’s Pharmaceutics: The Design and Manufacture of Medicines, 2013, 4th edition, Elsevier.

[24] Kumadoh D. , Adotey J. , Ofori-Kwakye K. , Kipo S. L. , Prah T. , and Patterson S. , Development of Oral Capsules From Enterica Herbal Decoction: A Traditional Remedy for Typhoid Fever in Ghana, Journal of Applied Pharmaceutical Science. (2015) 5, no. 4, 83–88.

[25] Gouissi D. H. A. , Nzangue R. T. , Kalaza J. H. , Pabo W. , and Chegaing S. P. F. , Medicinal Plants Used for Malaria Treatment in Gamba Village, North Region of Cameroon: Ethnopharmacological Survey, In Vivo Antimalarial Activity of Aqueous Extracts of Khaya Senegalensis Bark. (2021) .

[26] Aladesanmi A. J. , Awe S. O. , Adesanya S. A. , and Bray D. H. , Antimalarial Activity of Some Nigerian Medicinal Plants, Drug Production from Natural Products, Proceedings of the Seventh International Symposium on Medicinal Plants, 1988, Medex Publications Ltd..

[27] Akin-Osanaiya B. C. , Nok A. J. , Ibrahim S. et al., Antimalarial Effect of Neem Leaf and Neem Stem Bark Extracts on *Plasmodium berghei* Infected in the Pathology and Treatment of Malaria, International Journal of Research in Biochemistry and Biophysics. (2013) 3, no. 1, 7–14.

[28] Cudjoe E. , Donu D. , Okonu R. E. , Amponsah J. A. , and Amoah L. E. , The in Vitro Antiplasmodial Activities of Aqueous Extracts of Selected Ghanaian Herbal Plants, Journal of Parasitology Research. (2020) 2020, 1–8, 10.1155/2020/5041919. PMC7256685 32518693

[29] Coimbra E. S. , Santos J. A. , Lima L. L. et al., Synthesis, Antitubercular and Leishmanicidal Evaluation of Resveratrol Analogues, Journal of the Brazilian Chemical Society. (2016) 27, 2161–2169, 10.5935/0103-5053.20160107, 2-s2.0-84999633636.

[30] Saedi Dezaki E. , Mahmoudvand H. , Sharififar F. , Fallahi S. , Monzote L. , and Ezatkhah F. , Chemical Composition Along With Anti-Leishmanial and Cytotoxic Activity of Zataria Multiflora, Pharmaceutical Biology. (2016) 54, no. 5, 752–758, 10.3109/13880209.2015.1079223, 2-s2.0-84960451733. 26449681

